# Does Low-Magnitude High-Frequency Vibration (LMHFV) Worth for Clinical Trial on Dental Implant? A Systematic Review and Meta-Analysis on Animal Studies

**DOI:** 10.3389/fbioe.2021.626892

**Published:** 2021-04-27

**Authors:** Xinjian Ye, Ying Gu, Yijing Bai, Siqi Xia, Yujia Zhang, Yuwei Lou, Yuchi Zhu, Yuwei Dai, James Kit-Hon Tsoi, Shuhua Wang

**Affiliations:** ^1^School of Stomatology, Zhejiang Chinese Medical University, Hangzhou, China; ^2^Applied Oral Sciences and Community Dental Care, Faculty of Dentistry, The University of Hong Kong, Pokfulam, Hong Kong; ^3^The First Clinical Medical College, Zhejiang Chinese Medical University, Hangzhou, China; ^4^Hospital of Stomatology, Zhejiang Chinese Medical University, Hangzhou, China

**Keywords:** low-magnitude high-frequency vibration, osseointegration, osteoporosis, systematic review and meta-analysis, dental implant

## Abstract

Being as a non-pharmacological medical intervention, low-magnitude high-frequency vibration (LMHFV) has shown a positive effect on bone induction and remodeling for various muscle diseases in animal studies, among which dental implants osteointegration were reported to be improved as well. However, whether LMHFV can be clinically used in dental implant is still unknown. In this study, efficacy, parameters and side effects of LMHFV were analyzed via data before 15th July 2020, collecting from MEDLINE/PubMed, Embase, Ovid and Cochrane Library databases. In the screened 1,742 abstracts and 45 articles, 15 animal studies involving 972 implants were included. SYRCLE's tool was performed to assess the possible risk of bias for each study. The GRADE approach was applied to evaluate the quality of evidence. Random effects meta-analysis detected statistically significant in total BIC (*P* < 0.0001) and BV/TV (*P* = 0.001) upon loading LMHFV on implants. To conclude, LMHFV played an active role on BIC and BV/TV data according to the GRADE analysis results (medium and low quality of evidence). This might illustrate LMHFV to be a worthy way in improving osseointegration clinically, especially for osteoporosis.

**Systematic Review Registration:** https://www.crd.york.ac.uk/PROSPERO, identifier: NCT02612389

## Introduction

In recent years, oral implantation has become one of the favorable therapeutic strategies for wide range of dental application from simple missing tooth to reconstruction of edentulous jaw (Liu et al., 2017). A tight biological connection between bone tissue and embedded implant, also known as osseointegration, is considered as the foundation of clinical implantation (Zhang et al., 2020). Generally speaking, firm osseointegration is essential to improve implant stability in its early phase and long-term success rate (Simmons et al., 1999; Romanos, 2004). Nowadays, immediate implantation has been raised to realized implant insert with coinstantaneous loading (Tuminelli et al., 2017). Although similar survival rate was reported on immediate and delayed protocol, immediate restoration was limited by the immature early osseointegration (Chen et al., 2019). Thus, clinical failures owing to unstable osseointegration were also occasionally happened (Sanz-Sánchez et al., 2015).

In the meantime, some patients are suffering from metabolic bone diseases such as osteoporosis, which is one of the systemic risk factors for implant failure (Armas and Recker, 2012; Jiang and Xia, 2018). The difficulties for osteoporosis implantation were reported to be poor bone mass and bad wound recovery, which might lead to a pessimistic implant success rate (Cochran et al., 2011; Aghaloo et al., 2019). More seriously, an improper treatment has a potential harm on the bone healing time and implant osseointegration after surgery. Therefore, a careful, comprehensive and effective method is urgently required to promote implant osseointegration in these medically compromised patients (Aghaloo et al., 2019; Castellanos-Cosano et al., 2019).

It is well-known that the bone is able to adjust its mass and micro-structure upon biomechanical loading (Savoldi et al., 2017, 2018a). LMHFV is the vibration wave triggered by low-magnitude and high-frequency that elicits a positive effect on bone induction and remodeling (Lau et al., 2010; Thompson et al., 2015). LMHFV can be classified into whole-body vibration (WBV) and direct-loading vibration (DLV). For WBV, a biomechanical loading is acted on the whole body through trunk to apply an indirect force on the implant. For DLV, a biomechanical loading is acted on the specific part as needed, aiming to create a direct coupling on the implant (Zhang et al., 2012a; Corbiere and Koh, 2020). As a non-pharmacological intervention, LMHFV has been widely applied in the field of skeletal muscle diseases for its simple, convenient, non-invasive and early-effective characteristics (Judex et al., 2007; Holguin et al., 2009). Notably, its application potential in oral implant surgeries is still emerging. Previous studies (Rubin et al., 2001; Jing et al., 2015, 2018) have reported that LMHFV could stimulate bone healing and osseointegration on peri-implant bones. Although increasing evidences have shown the correlation between LMHFV and osseointegration, the defined outcome and results remain unclear. In the present study, a prospective systematic review and meta-analysis on animal studies is conducted as a pre-clinical screening to analyze the parameters, efficacy, and adverse effects of LMHFV on osseointegration so as to provide useful references in the application of clinical trials.

## Methods

### Study Design

This systematic review and meta-analysis was registered in the international prospective register of systematic reviews (PROSPERO) (CRD42020200276) following the preferred reporting items for systematic review and meta-analysis (PRISMA) statement and the population, interventions, comparisons, outcomes, study design (PICOS) question to these investigations (Moher et al., 2015; Shamseer et al., 2015):

- Population: Animals (ovariectomized, non-ovariectomized);
- Interventions: LMHFV (WBV, DLV) loading;
- Comparisons: Sham-loading or non-loading;
- Outcomes: BIC, BV/TV (primary outcomes); parameters, gene expressions, adverse effects and relevant study outcomes (secondary outcomes);
- Study design: RCT studies.

The focused question was “Does LMHFV enhance the implant osseointegration in animal models?” Appendix 1 shows the specific screening criteria based on the PICOS question.

### Classification of Outcome Measures

**Primary outcome measures:** The assessed primary outcomes were bone-to-implant contact (BIC) and peri-implant bone volume relative to tissue volume (BV/TV), which were defined as follows:

(1) BIC, %: the sum of bone contact region lengths with implant (mm) divided by total length along the implant from the first to the last (mm);
(2) BV/TV, %: the amount of bone within a specific region of interest (mm^2^) divided by the total amount of bone on the implant surface (mm^2^).

**Secondary outcome measures:** Secondary outcomes included parameter (magnitude, frequency, duration), gene expression and adverse effect of LMHFV treatment.

### Search Strategy

An electronic search was conducted among four databases (MEDLINE/PubMed, Embase, Ovid and Cochrane Library) from inception until 15th July 2020. The search was not limited by any restrictions on language or publication. Each database search combines concepts and subject headings, as detailed in Appendix 2.

An extensive manual search was also performed through included references and other related systematic reviews. In addition, relevant articles published between July 2000 and July 2020 were also inspected. The journal list was depicted as outlined in Appendix 3. Finally, the “specific theses database” (www.theses.com) and the “Gray Literature” (opensigle.inist.fr) were additionally screened for ongoing studies.

### Search Criteria

**Inclusion criteria:** (1) RCTs of animals; (2) Ovariectomized (OVX) or non-ovariectomized (non-OVX) implant osseointegration model; (3) More than one sham-loading control group; (4) the effect of LMHFV on peri-implant bone healing or osseointegration process; (5) Definition of the LMHFV's parameters and application; (6) Mean bone-to-implant contact (BIC, %) and/or mean peri-implant bone volume relative to tissue volume (BV/TV, %); (7) Outcomes related to peri-implant bone morphology/efficacy/adverse effects/gene expressions; (8) Full-text literature published in English before 15, July, 2020.

**Exclusion criteria:** (1) Included parameters were not consistent with LMHFV definition; (2) Sample size was <3 animals; (3) Treatment period was <2 weeks; (4) Only positive control group was set; (5) Outcomes of the studies remained ambiguous or unavailable; (6) Not animal RCTs (e.g., clinical trials, cell investigations, cross-sectional studies, cohort studies); (7) Other implant osseointegration models of animals (e.g., diabetes mellitus models, bone defect models); (8) Other publication types (reviews, conference reports).

### Selection of Studies

Study selection was conducted by two trained researchers (XY and YB) independently. The reviewers received a professional training prior to the formal evaluation, and the level of agreement between the reviewers of screening was calculated by kappa statistics. All stages (titles, abstract, full-text) were carried out in duplicate to exclude irrelevant papers in initial screening. Then, relevant studies of full-text were retrieved and reviewed based on specific inclusion and exclusion criteria to confirm study eligibility. Study exclusion reasons were recorded as well. We resolved any disagreement by discussion with a third author (SW). Finally, in case of incomplete data, the corresponding authors of included studies were contacted and asked for further information.

### Data Extraction

Two reviewers (YZ and YZ) extracted the information independently. A third reviewer (XY) moderated any disagreement if needed. Extracted information on study characteristics were presented in Appendix 4. Data was synthesized in a data form which was specifically designed for meta-analysis according to significant category methods, and measurement data was carried out on *Microsoft Excel (version 16.38, Microsoft Corp, Redmond, US)*. For relevant missing data, we have attempted to contact original authors. Only if we failed to contact with authors in 3 times, we would use *GetData Graph Digitizer (version 2.26*, getdata-graph-digitizer.com, Germany) to obtain the data in the chart.

### Quality Assessment

In this study, the risk of bias was assessed by SYRCLE's tool (Hooijmans et al., 2014; Zeng et al., 2015), while the level of evidence was assessed by GRADE approach *(GradeProSetup, the Cochrane Collaboration*) (Guyatt et al., 2008; Balshem et al., 2011). A calibration exercise was conducted before the assessment in order to ensure consistency across reviewers. Judgements were made independently by two reviewers (SX and YD) based on the criteria for judging the risk of bias. Disagreements were resolved by discussion and consulting a third author (YG) for arbitration and consensus. Extracted data were combined into a summary *Excel (version 16.38, Microsoft Corp, Redmond, US)*.

### Statistical Analysis

A systematic narrative summary was provided with information presented in table to summarize and clarify included studies' characteristics. The data was collected manually into three subgroups (OVX-WBV, non-OVX-WBV, and non-OVX-DLV). The I^2^ statistics was used to assess the statistical heterogeneity in each subgroup. We considered heterogeneity to be statistically significant if *P* < 0.1 (Du et al., 2020). Due to the statistical heterogeneity, the random effect model was chosen for meta-analysis by *RevMan (version 5.3, RevMan, the Cochrane Collaboration)*. Continuous outcomes (BIC and BV/TV) were analyzed by using standardized mean difference (SMD) *w*ith 95% confidence intervals (CI), *P* < 0.05 was set as significance. Finally, the analysis of the effect on parameters was presented in bubble chart using *R package ggplot2 (version 3.3.2*, cran.r-project.org).

## Results

### Results of the Search

**Figure 1:**
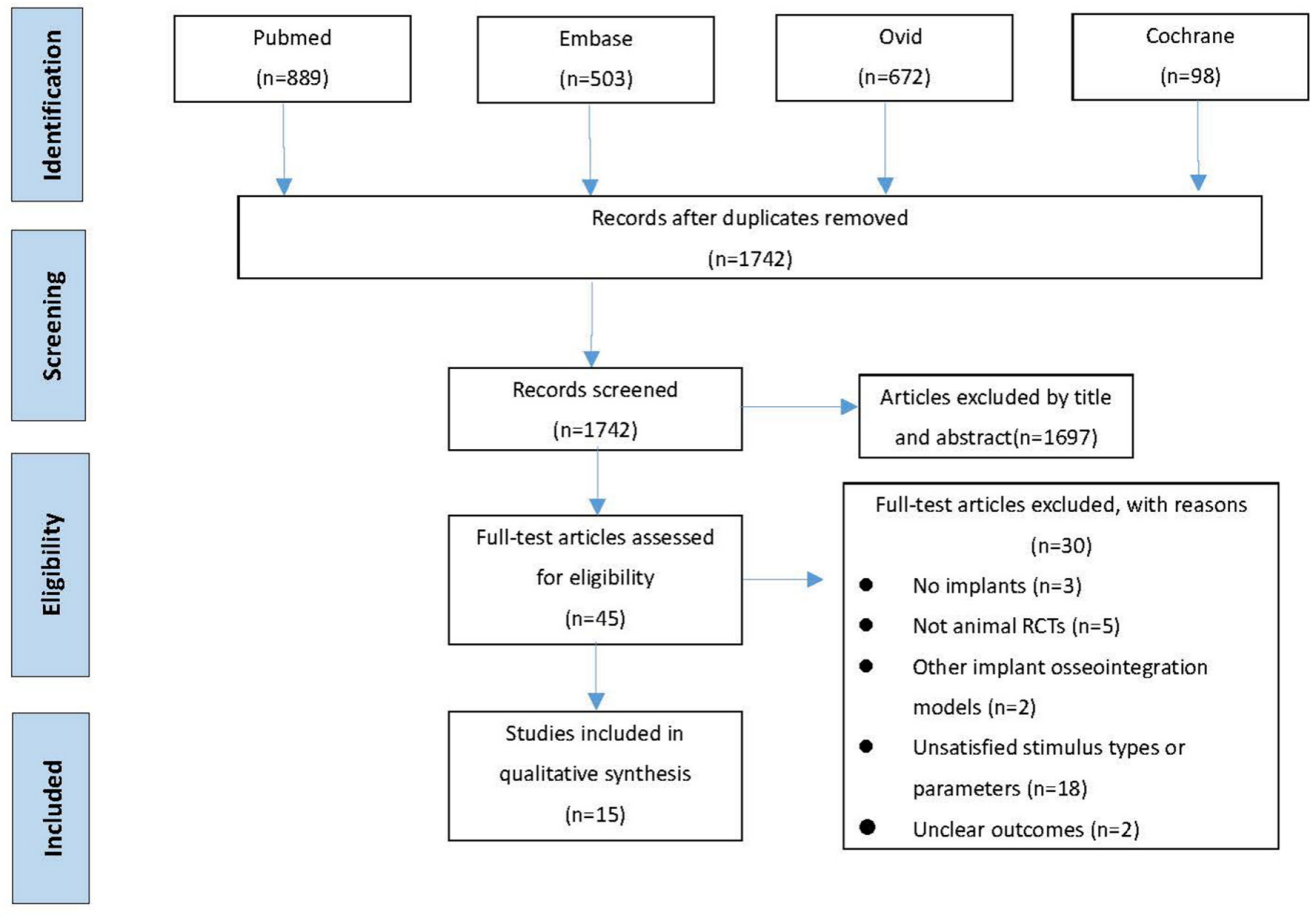
PRISMA flowchart of the screening process.

In this systematic review and meta-analysis, 2,162 studies were searched and selected from MEDLINE/PubMed database (889 studies), Embase database (503 studies), Ovid database (672 studies), and Cochrane Library database (98 studies). After duplicates removed from 2,162 studies, 1,741 studies remained for further analysis. Based on rough evaluation on abstracts, unsatisfied 1,697 studies were excluded due to their topic or content. Afterwards, 30 studies (Figure 1) were left under rigorous evaluation on remained 45 full articles. The most common reasons for exclusion were unsatisfied stimulus types, unacceptable stimulus parameters or absence of animal RCTs. The reasons for excluded articles were recorded detailly in Appendix 1. Besides, no qualified article was found via manual search. Eventually, 15 manuscripts (Akca et al., 2007; Shi et al., 2010; Ogawa et al., 2011a,b, 2014; Chen et al., 2012; Zhang et al., 2012b,c; Chatterjee et al., 2014; Liang et al., 2014; Zhou et al., 2015; Wang et al., 2016, 2018; Ruppert et al., 2018, 2019; Shibamoto et al., 2018) were recruited for qualitative analysis.

### Characteristics of the Included Studies

Descriptive characteristics of included studies were listed in Table 1. Fifteen selected studies were conducted between 2007 and 2019, including 13 rat studies (6 OVX rat studies and 7 non-OVX studies), 1 rabbit study, and 1 dog study. Among them, 13 studies reported tibia implantation, 1 study revealed femur implantation, while only 1 beagle dog study conducted implant surgical in mandibular bone. In terms of loading modes, 10 studies reported the effect of WBV and the others applied DLV instead. Three studies combined the effect of LMHFV and drugs, in which 2 studies explored the differences and connections due to different biomechanical types. In total, 811 animals, including 785 rats, 20 rabbits, and 6 dogs, were involved in this paper. The implant number is 1,096 in 811 animal studies, we selected 972 for this review.

**Table 1:** Summary of the study characteristic.

**References**	**Model**			**Animal**				**Implant**					**F-Up**	**LMHFV**		**Index**	
	**OVX**	**non-OVX**	**sham-OVX**	**N**	**type**	**sex**	**age**	**n**^**a**^/**n**^**b**^	**type**^c^	**Ø (mm)**	**L (mm)**	**Local**		**WBV**	**DLV**	**BIC**	**BV/TV**
Akca et al. (2007)	√	×	×	15	r	f	12 w	30/20	Ti	1	5	t	2 w	√	×	×	√
Chatterjee et al. (2014)	√	×	√	59	r	f	12 w	59/40	Ti	2	8	t	4–14 d	√	×	√	√
Chen et al. (2012)	√	×	√	40	r	f	5 mh	40/30	HA-Ti	1	10	t	8 w	√	×	√	√
Liang et al. (2014)	√	×	√	36	r	f	12 w	72/72	Ti	2	7	t	4 w	√	×	√	√
Shibamoto et al. (2018)	√	×	×	44	r	f	11 w	44/16	Ti	2	13	t	4 w	√	×	√	√
Zhou et al. (2015)	√	×	√	40	r	f	3 mh	80/80	HA-Ti	1	10	t	12 w	√	×	×	√
Ogawa et al. (2011a)	×	√	×	95	r	m	3 mh	95/92	Ti	2	10	t	1–4 w	√	×	√	√
Ogawa et al. (2011b)	×	√	×	42	r	m	A	42/40	Ti	2	10	t	3–25 d	√	×	√	√
Ogawa et al. (2014)	×	√	×	120	r	m	3 mh	120/119	Ti	2	10	t	1–4 w	√	×	√	√
Ruppert et al. (2018)	×	√	×	80	r	f	24 w	80/80	Ti6Al4V	2	10	t	6 w	√	×	√	√
Ruppert et al. (2019)	×	√	×	70	r	f	24 w	70/40	Ti6Al4V	1.5	20	fe	4–8 w	×	√	√	√
Wang et al. (2016)	×	√	×	20	rb	m	6 mh	40/40	Ti	1.2	9.25	t	20 d	×	√	×	√
Wang et al. (2018)	×	√	×	6	dg	m	3 mh	36/36	Ti	3.3	8	j	2–8 w	×	√	√	√
Zhang et al. (2012a)	×	√	×	69	r	m	A	138/131	Ti	2	8	t	29 d	×	√	√	√
Zhang et al. (2012b)	×	√	×	75	r	m	3 mh	150/123	Ti	2	10	t	1–4 w	×	√	√	×

a *Total number of implants included in the investigation*.

b *Total number of implants extracted for the review*.

c *Some studies included multiple types of surface treatments*.

*Animal type: r, rat; dg, dog; rb, rabbit; sex: f, female; m, male; age: A, adult; d, days; mh, months; w, weeks; local: fe, femur; j, jaw; t, tibia; Ø, diameter; BIC, bone-to-implant contact; BV/TV, volume/total volume; DLV, direct-loading vibration; F-Up, follow-up; HA, hydroxyapatite-coated; L, length; LMHFV, low-magnitude high-frequency vibration; Ti, titanium; WBV, whole-body vibration*.

## Outcomes

### Parameters

As a device-driven therapy, the parameters of LMHFV bear an important guiding significance for its application (Zhang et al., 2012b; Gao et al., 2016), which were defined in this paper as follows: Vibration magnitude <1 g (WBV) or 150 μm (DLV), frequency >20 Hz. According to different animal models and vibration patterns, three groups were classified, namely OVX-WBV group, non-OVX-WBV group, and non-OVX-DLV group.

Among studies involving non-OVX-WBV cases (Ogawa et al., 2011a,b, 2014; Ruppert et al., 2018), the frequency, magnitude, and weekly loading time ranged 45–140 Hz (MD: 85.0 ± 34.3 Hz), 0.043–0.6 g (MD: 0.28 ± 0.16 g), and 25–75 min (MD: 47.5 ± 22.2 min), respectively, which were 32.5–140 Hz (MD: 59.6 ± 40.0 Hz), 0.2–0.5 g (MD: 0.35 ± 0.12 g) and 70–300 min (MD: 142.2 ± 92.7 min), respectively in OVX-WBV subgroup (Chen et al., 2012; Chatterjee et al., 2014; Liang et al., 2014; Shibamoto et al., 2018). As OVX magnified defects in bone mass, the loading duration in OVX model was significantly longer than that in non-OVX model.

Generally, the amplitude (μm) was selected to replace DLV as its magnitude was often <0.1 g (Wang et al., 2016). The relationship among frequency, amplitude, and magnitude was expressed as follows:

(1) $$\begin{array}{l} \text{A}=\frac{9.81\text{g}}{{\left(2\pi \text{f}\right)}^{2}} \end{array}$$

where g (m/s^2^) represented the magnitude, A (m) was the amplitude, and f (Hz) was the frequency.

Among studies involving non-OVX-DLV cases (Shi et al., 2010; Zhang et al., 2012a,b; Wang et al., 2018; Ruppert et al., 2019), the frequency, the magnitude and weekly loading time ranged 20–100 Hz (MD: 51.0 ± 41.0 Hz), 7.7–73.8 μm (MD: 19.3 ± 19.2 μm), and 50–210 min (MD: 92.0 ± 62.5 min), respectively. In fact, the results from Equation (1) concluded that the values of magnitude and frequency of DLV were significantly lower than those of WBV treatment. However, the therapeutic effect of DLV was not as good as that of WBV, and lower levels of DLV parameters indicated worse outcomes. Three studies (Shi et al., 2010; Zhang et al., 2012b; Wang et al., 2018) explored the optimal vibration parameters of DLV treatment, and the best parameters were as follows: 20 Hz frequency, 15 μm magnitude, and 210 min weekly loading time in rabbits; 40 Hz frequency, 7.8 μm magnitude and 50 min weekly loading time in rats; 40 Hz frequency, 8 μm magnitude and 50 min weekly loading time in rats. Discrepancies between different parameters may be attribute to different animal models, types of implants, and loading sites.

**Figure 2:**
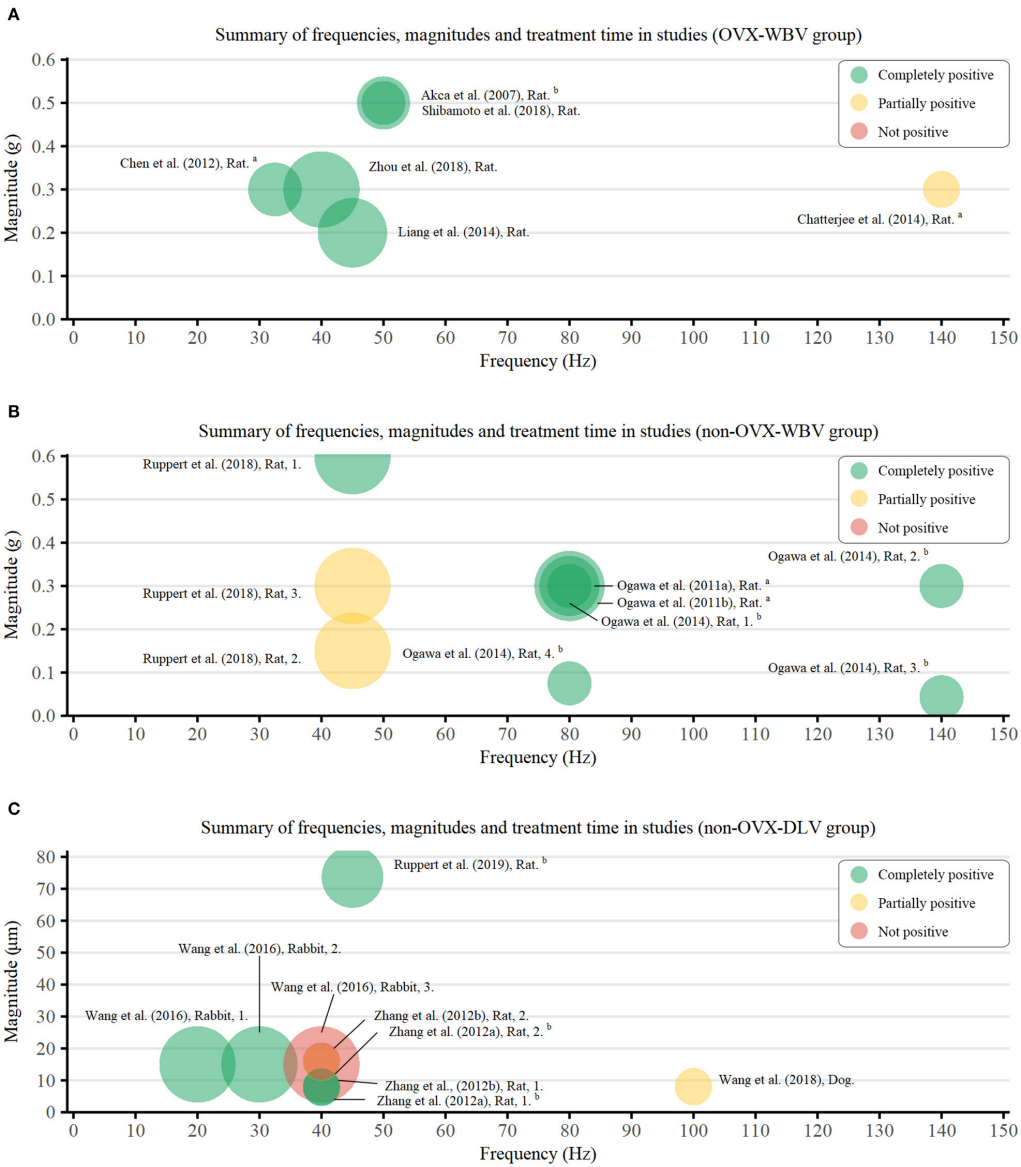
Bubble chart of frequencies (Hz), magnitudes (g/μm), and duration (min) of vibration treatment used in the studies. Green bubbles indicate more than two-thirds positive measurements, among which two test data were statistically significant (*P* < 0.05); Yellow bubbles indicate more than half positive measurements; Red bubbles indicate more than half negative measurements. Area of circle represents LMHFV loading duration. ^a^An average of the composite vibration; ^b^converted from other units. **(A)** OVX-WBV group; **(B)** non-OVX-WBV group; **(C)** non-OVX-DLV group.

To clarify the relationship between parameters and effect of LMHFV, we extracted eligible data and results of LMHFV from each study and plotted them into Figure 2. Relevant parameters from a total of 24 groups were extracted from 15 studies, including 6 OVX-WBV group, 9 non-OVX-WBV group, and 9 non-OVX-DLV group. There were 5 and 7 studies demonstrated beneficial effects of WBV on osteointegration enhanced by vibration treatment in OVX animals and non-OVX animals, respectively. One showed that a relative high frequency in early period do not have a significant positive effect on OVX rats, and 2 showed that a relative low frequency with a low magnitude does not have a significant positive effect on non-OVX rats. For 9 studies reporting the effect of DLV on non-OVX animals, 5 showed positive effects on rabbits and rats, and the remaining showed no significant or no positive influence of vibration during osseointegration.

### Meta-Analysis

Complexities of pre-clinical researches (animal species, experiment methods etc.) might result in meta-analysis heterogeneity. Hence, subgroup analyses were conducted to eliminate possible sources of heterogeneity. Due to different experimental design, the extracted data were mostly close to the following parameters: 50 Hz, 0.3 g/8 μm, 2 w, medulla.

#### BIC

**Figure 3:**
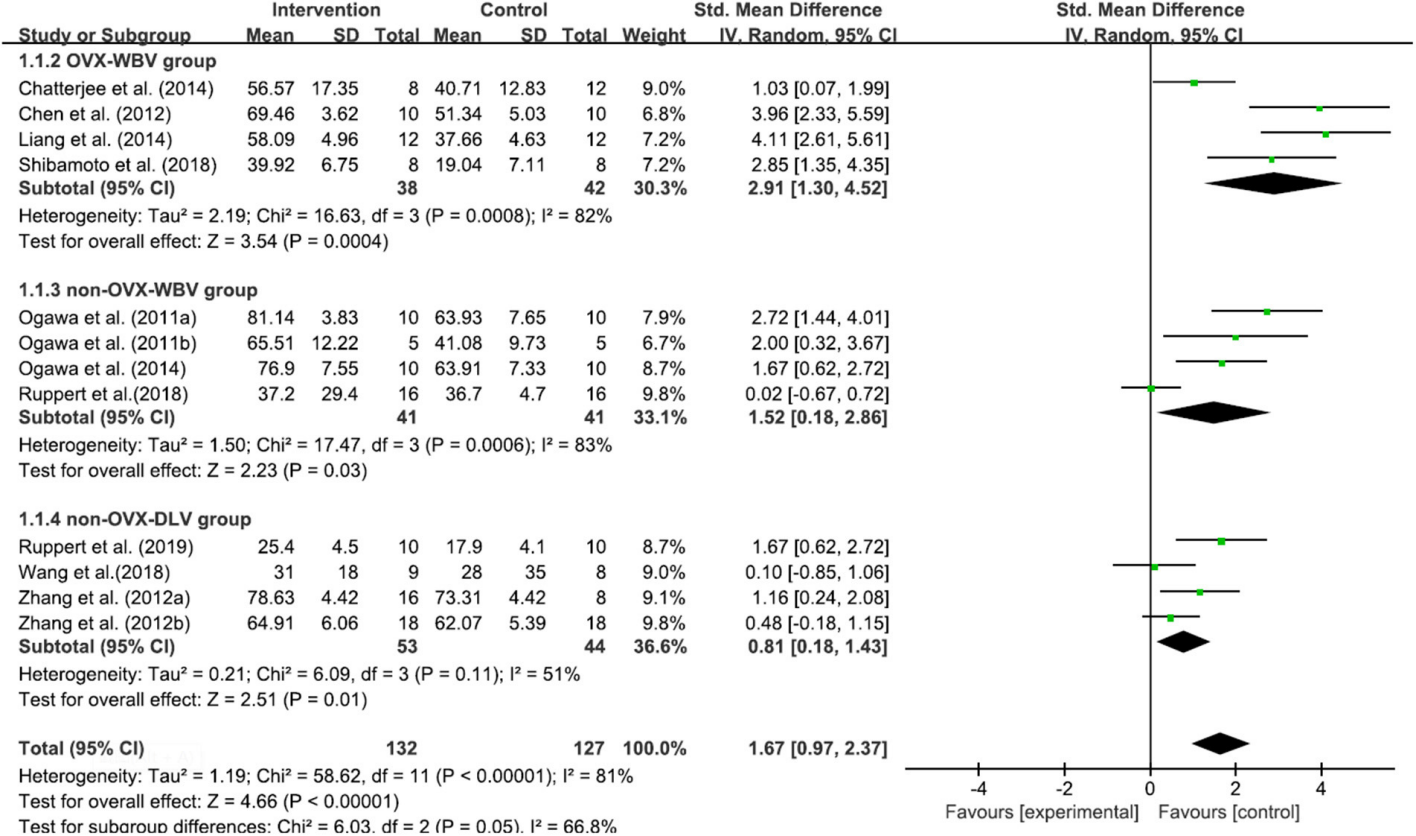
Meta-analysis. BIC Standardized mean differences of LMHFV treated compared to sham or untreated animals.

Twelve included studies (Shi et al., 2010; Ogawa et al., 2011a,b, 2014; Chen et al., 2012; Zhang et al., 2012b,c; Chatterjee et al., 2014; Liang et al., 2014; Ruppert et al., 2018, 2019; Shibamoto et al., 2018; Wang et al., 2018) contributed data for analysis from 259 subjects, and there was a high statistical heterogeneity among the studies (*P* < 0.00001, *I*^2^ = 81%). The random-effects model was used for meta-analysis. According to the results, the BIC of the test group was higher than that of the control group [MD = 1.67, 95% CI (0.97, 2.37), *P* < 0.00001]. All of the 3 subgroups, that is, OVX-WBV [MD = 2.91, 95% CI (1.30, 4.52), *P* = 0.0004] (Chen et al., 2012; Chatterjee et al., 2014; Liang et al., 2014; Shibamoto et al., 2018), non-OVX-WBV [MD = 1.52, 95% CI (0.18, 2.86), P=0.03] (Ogawa et al., 2011a,b, 2014; Ruppert et al., 2019) and non-OVX-DLV [MD = 0.81, 95% CI (0.18, 1.43), *P* = 0.01] (Shi et al., 2010; Zhang et al., 2012a; Wang et al., 2018; Ruppert et al., 2019) showed statistically significant differences (Figure 3).

#### BV/TV

**Figure 4:**
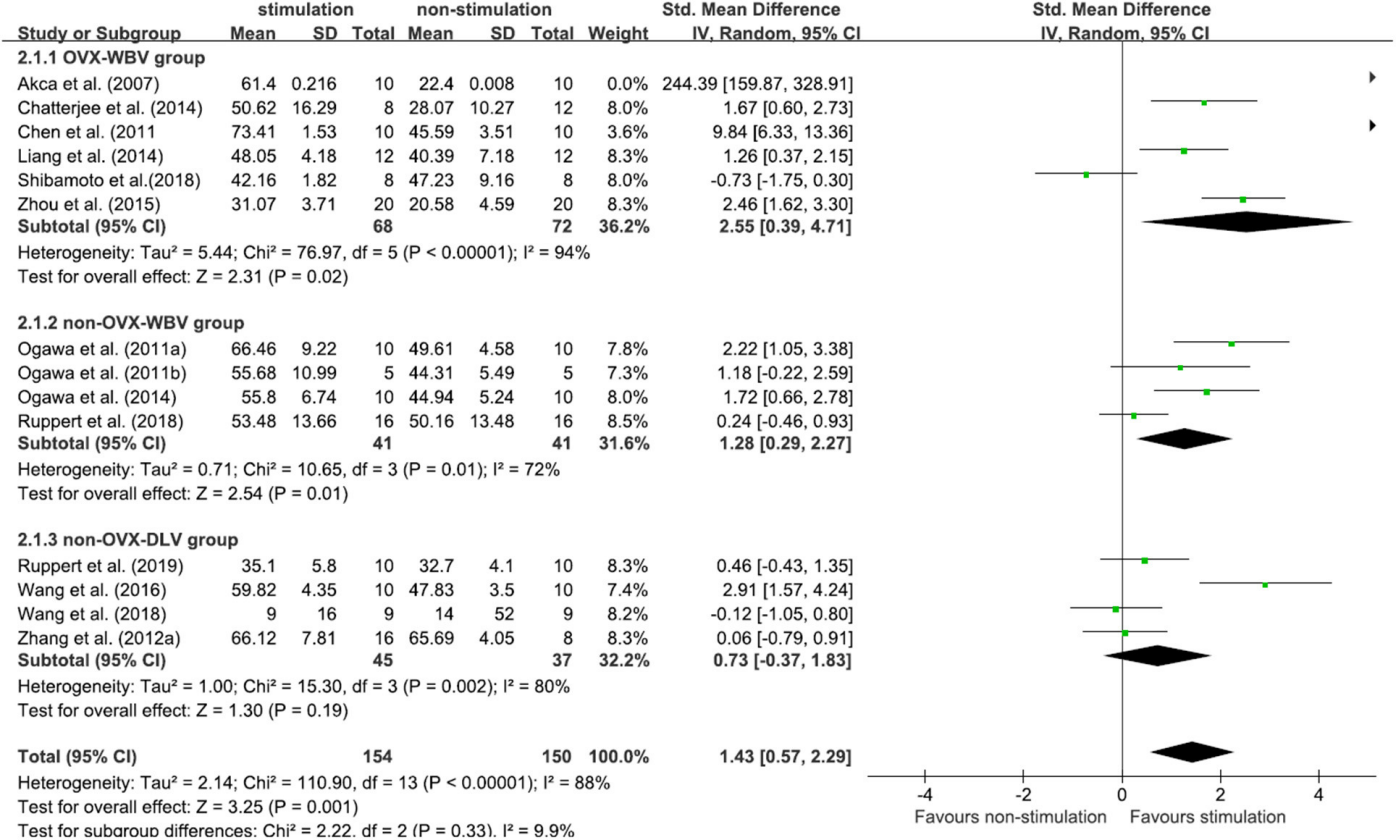
Meta-analysis. BV/TV Standardized mean differences of LMHFV treated compared to sham or untreated animals.

Fourteen included studies (Akca et al., 2007; Shi et al., 2010; Ogawa et al., 2011a,b, 2014; Chen et al., 2012; Chatterjee et al., 2014; Liang et al., 2014; Zhou et al., 2015; Wang et al., 2016, 2018; Ruppert et al., 2018, 2019; Shibamoto et al., 2018) contributed data for analysis from 304 subjects, and there was a considerable statistical heterogeneity among the studies (*P* < 0.00001, *I*^2^ = 88%). The random-effects model was used for meta-analysis. According to the results, the BV/TV of the test group was higher than that of the control group, and the difference was statistically significant [MD = 1.43, 95% CI (0.57, 2.29), P < 0.00001]. Among the three subgroups, OVX-WBV [MD = 2.55, 95% CI (0.39, 4.71), *P* = 0.02] (Akca et al., 2007; Chen et al., 2012; Chatterjee et al., 2014; Liang et al., 2014; Zhou et al., 2015; Shibamoto et al., 2018) and non-OVX-WBV [MD = 1.28, 95% CI (0.29, 2.27), *P* = 0.01] (Ogawa et al., 2011a,b; Ogawa et al., 2014; Ruppert et al., 2018) showed statistically significant benefits, non-OVX-DLV [MD = 0.73, 95% CI (−0.37, 1.83), *P* = 0.19] (Shi et al., 2010; Wang et al., 2016, 2018; Ruppert et al., 2019) demonstrated benefits for test groups but not significant (Figure 4).

### Qualitative Analyze

Table 2 listed the main findings about LMHFV/osseointegration/conclusion of the recruited eligible studies. Though lack of quantitative analyze, the effect of LMHFV on gene expression and adverse effect were discussed as follows:

**Table 2:** The impact of LMHFV on osseointegration.

**References**	**Results I** **(Findings related to osseointegration)**	**Results II** **(Findings related to LMHFV)**	**Conclusion**
Akca et al. (2007)	• The BV/TV of peri-implant bone was increased in experiment group. • WBV can't affect Tb.Th and Tb.Sp	• WBV (50 Hz, 5 N, 2 w) performed more effective than PEMFs	• Biophysical stimulation remarkably enhances bone volume around titanium implants placed in OVX rats
Chatterjee et al. (2014)	• WBV significantly increased BIC at medullary levels, but cortical bone around the implant is less sensitive to HF-WBV loading	• The combination of ALN and HF-WBV didn't affect the bone healing response	• In the case of osteoporosis, HF-WBV has a positive effect on implant osseointegration
Chen et al. (2012)	• The BIC, BF and BMD of OVX-WBV group were higher than those of OVX group, but lower than that of sham-OVX group and OVX-ALN group	• The combination of the ALN and WBV (30–35 Hz, 0.3 g, 8 w) may have a synergistic or cumulative effect on osteoporosis patients	• WBV enhanced bone-implant osseointegration in OVX rats, but it was not as effective as ALN
Liang et al. (2014)	• The BIC, BF, MAR and TBL of OVX-WBV group were higher than those of OVX group, but lower than that of sham-OVX group	• NA	• Four-week WBV loading reduced the negative effects of osteoporosis and promoted bone healing around implants in OVX rats
Shibamoto et al. (2018)	• Compared with the control group, BIC and BV/TV were increased in all WBV loading group	• WBV (50 Hz, 0.5 g, 4 w) loading and PTH have additive effects on peri-implant bone healing and osseointegration in OVX rats	• LMHF loading and PTH can act locally and additively on the bone healing process, improving the condition of implant osseointegration
Zhou et al. (2015)	• In OVX-WBV group, BV/TV, Conn, Tb.N, Tb.Th were increased, and Tb.Sp were decreased. • WBV-driven genes and proteins including Runx2, OPN, OC, RANKL, M-CSF and OPG to facilitate bone formation rather than bone resorption	• NA	• WBV has been shown to be beneficial in improving osseointegration in osteoporotic condition. • Activation of ERK1/2 plays an important role in vibration-induced bone remodeling
Ogawa et al. (2011a)	• The BIC and BF was significantly increased in the experimental group	• Twice 1.25 min of loading seemed to have the most favorable effect	• LMHF loading with a particular time sequence can stimulate peri-implant bone healing and formation
Ogawa et al. (2011b)	• LMHF loading increased BIC and BF significantly.	• The loading effect seemed to decrease as the distance to the implant increased.	• LMHF loading had a bone-stimulating potential, through WBV, on peri-implant bone healing and implant osseointegration
Ogawa et al. (2014)	• The BIC and BV/TV was increased in the experimental group.	• Loading regimes at high acceleration with medium or high frequency (70-90/130-150 Hz, 0.3 g, 4 w) showed significant results	• Highfrequency vibration loading showed potential to accelerate and enhance implant osseointegration
Ruppert et al. (2018)	• The BV/TV and bone volume around the implant was significantly improved after WBV treatment	• Amplitude at 45 Hz, 0.6 g, 5 w seemed to showed the most favorable effect. • There was no significant difference between different amplitudes	• Vibration has demonstrated its therapeutic benefits for increasing bone adjacent to the implant
Ruppert et al. (2019)	• DLV demonstrated improved peri-implant bone volume with increased BIC, but showed no statistical difference	• LIPUS was superior to vibration for accelerating osseointegration at 4 weeks	• Vibration has demonstrated its therapeutic benefits for increasing bone adjacent to the implant
Wang et al. (2016)	• The BIC, BV/TV and the number of osteoclasts was increased in the DLV group	• DLV at 20 Hz, 15 μm, 20 d result in signifificant improvement, however, the use of 40 Hz did not. • Temporary weight loss were noticed on the experimental animals	• The application of a direct LMHF (10, 20, or 30 Hz) micro-vibration on implants promoted bone formation and osseointegration
Wang et al. (2018)	• The treatment group had significantly increased in BIC compared with the control group after 2 weeks of loading	• There was no significant difference between groups after 8 weeks	• DLV loading positively influenced peri-implant bone healing in the early healing period
Zhang et al. (2012c)	• BIC was inhibited in the experimental group. • Normal healing response after implantation was irrespective of the loading regime. • The expression of calcitonin gene was increased in the experimental group	• Significant increase was only found in case of HF-LM loading (40 Hz, 0.5 N, 4 w). • The peri-implant tissue response to the loading via different modes of application varies. WBV seems to be superior to DLV	• Mechanical loading contributed to peri-implant bone healing. • To provoke a positive response, LF loading required higher magnitudes than HF loading
Zhang et al. (2012b)	• The BIC increased at cortical and medullary compared to the control group. • The BF did not significantly increase after loading	• Significant increase was only found in case of HF-LM loading (40 Hz, 8 mm, 4 w) at cortical. • The applied load regimes failed to influence the peri-implant bone mass	• The effect of implant loading on bone-to-implant contact was only observed in case of high-frequency low-magnitude loading

*ALN, alendrinate; BF, bone fraction; BIC, bone-to-implant contact; BMD, bone mineral density; BV/TV, bone volume/total volume; Conn, connectivity density; DLV, direct-loading vibration; LIPUS, low-intensity pulsed ultrasound; LMHFV, low-magnitude high-frequency vibration; OVX, ovariectomized; PEMF, pulsed electromagnetic field; PTH, parathytoid hormonr; Tb.N, trabeculae number; Tb.Sp, trabecular separation; Tb.Th, trabecular thickness; WBV, whole-body vibration*.

#### Gene Expression

Generally speaking, the bone transforms mechanical signals into biological signals, and further regulates osteoblast lineages and cartilage formation of mesenchyhmal stem cells through adjustment factors (Rubin et al., 2000; Fan et al., 2006). Two included studies (Shi et al., 2010; Zhou et al., 2015) reported expression levels of genes associated with LMHFV. As a result, osteogenesis-associated genes were up-regulated after loading, while those associated with osteoclast generation were down-regulated (Lau et al., 2010). Adjustment factors (such as BMP2, ALP, OCN, Runx2, Wnt3a, Lrp6, β-catenin, Sost, RANKL, OPN, OC, M-CSF, and OPG) were involved in this process (Lau et al., 2010; Birmingham et al., 2015; Ota et al., 2016). In addition, the Wnt/β-catenin, RANKL and MAPK-ERK 1/2 signaling pathways participate in the process of osteoblast behaviors (Pichler et al., 2013; Li et al., 2015; Zhou et al., 2015; Chen et al., 2016).

#### Adverse Effect

Since the curative effect of LMHFV has been affirmed, the safety of its application has been well-concerned. Among fifteen included studies, no serious adverse events of LMHFV were reported in any included studies. One study (Wang et al., 2016) reported a temporary weight loss on experimental animals, whilst all animals well-tolerated this process. In general, a short-term application of LMHFV was relatively safe, and a constant or intense application of LMHFV may slightly influence physical functional metabolism (Branemark et al., 2014; Ruppert et al., 2019). One study (Xie et al., 2016) indicated that a long-term WBV loading might contribute to bone trabeculae loss in OVX rats and aggravate osteoporosis. Besides, loading parameters also exert an independent role. For example, compared to continuous loading, cyclic loading mitigates the development of tolerance and maintains a high efficacy (Umemura et al., 2002). In comparison to vertical loading, a composite loading reduces the intensity of the stimulus and alleviates the adverse effects of treatment (Torvinen et al., 2003).

### Quality Assessment

**Figure 5:**
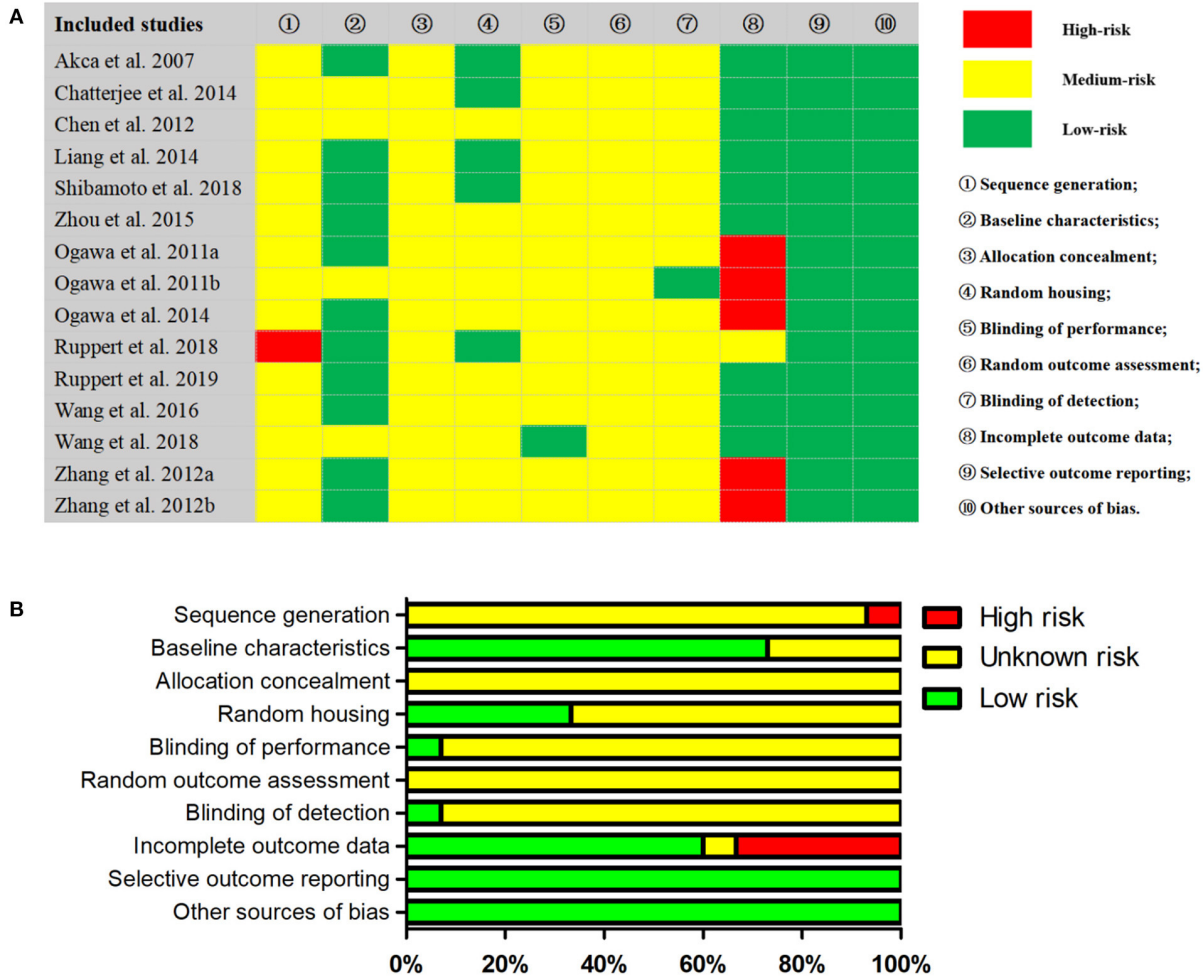
Results of SYRCLE's RoB tool in included studies. **(A)** Representative summary table for the risk of bias assessment; **(B)** Representative summary for risk of bias analysis across studies.

The kappa test result of the two evaluators was 0.74, which was considered as qualified. Following the standards of SYRCLE's tool, a total of 10 items were evaluated (Figure 5). Specifically, one study (Ruppert et al., 2018) was identified as high risk of sequence generation because there was no clear indication of randomization. Five studies (Ogawa et al., 2011a,b, 2014; Zhang et al., 2012b,c; Wang et al., 2018) were identified as high risk of incomplete outcome data because there existed some missing sample data without an adequate explanation. However, many items were not explained in detail in the included studies, which may lead to great difficulties and deviations in the interpretation of research bias. The GRADE approach was performed to assess the level of evidence body. In detail, the evidence quality of two BIC subgroups (OVX-WBV, non-OVX-DLV) and one BV/TV subgroup (OVX-WBV) were moderate level, while the others were low (Appendix 6).

## Discussion

To the best of our knowledge, this was the first prospective systematic review and meta-analysis about LMHFV on osseointegration, which comprehensively discussed the efficacy, gene expressions and adverse effects in animal models, and further analyzed osseointegration with regard to specific loading regimens.

### Summary of Key Findings

Focusing on the study results alone, the positive effect of WBV treatment was more definite than that of DLV treatment. Meta-analysis showed that both WBV (*P* = 0.0006) and DLV (*P* = 0.01) have positive effects on improving BIC in non-OVX groups. However, WBV (*P* = 0.01) presented a stronger effect on osteogenesis in comparison to DLV (*P* = 0.19), manifesting as the improved BV/TV. It is believed that the weak effect of DLV treatment may result in an insufficient loading transfer to surrounding tissues or a massive micro-interface movement, further leading to an insufficient force conduction and a sparse embedding of soft tissues (Shi et al., 2010; Zhang et al., 2012b,c).

In addition, the osteogenic effect of LMHFV was more significant on OVX animal models. Since osteoporosis affects the quality and quantity of whole-body bones, WBV is more suitable for treatment (Marín-Cascales et al., 2018), while DLV has more direct efficiency on peri implant osseointegration. Meta-analysis showed that WBV could improve BIC in both non-OVX-WBV (*P* = 0.03) and non-OVX-DLV (*P* = 0.0004) groups. Also, BV/TV was comparable between groups because WBV caused bone growth in both non-OVX (*P* = 0.01) and OVX (*P* = 0.02) models. Due to bone mass reduction and bone structure deterioration, the loss of the initial stability of implants in osteoporosis patients is likely to cause an implant failure without a proper treatment (Friberg et al., 2001). The evidence of this meta-analysis suggested that LMHFV could reverse the negative effect caused by osteoporosis partially and enhance bone healing and osseointegration in peri-implant tissues.

### Quality of the Evidence

The GRADE approach showed that three subgroups (non-OVX-DLV in BV/TV, non-OVX-WBV in BIC and BV/TV) were not conclusive. This might be high risk of bias and considerable heterogeneity within one subgroup. Despite less influence on our result, the evidence was low quality, which needs to be with caution.

In fact, in medical field, systematic reviews are one of the powerful keys to provide high level of evidence (Albelasy et al., 2020; Pound and Ritskes-Hoitinga, 2020), that is essential and formed a basis for pre-clinical assessment for medical devices (Savoldi et al., 2018b) and drugs (Faggion, 2015). Nonetheless, animal trial design in particular RCT, is important because a proper design signifies the success of the translational outcome (Hall and Traystman, 2009; Mak et al., 2014). Thus, this research only selected RCT trials on animal studies. It should be noticed that animal study cannot replace or be a guide for any human clinical trial (Van Norman, 2020), but rather a proof for authority to approve a certain level of safety (Pound et al., 2004). Indeed, all experiments might have certain limitations and it is important to report all factors, no matter contributive or destructive, so that readers or even the “artificial intelligence” can learn the facts genuinely. For the published data, prospective systematic review on animal studies was indeed recently advocated that can produce sound and actual clinical knowledge (Pound and Ritskes-Hoitinga, 2020), under the circumstances proper designs and valid models which can be found in methotrexate (Leenaars et al., 2020) and dental implant (Manzano et al., 2014) studies that were with meta-analysis. Thus, our current study can give a frank, fair, and open opinion to all readers.

### Further Observations

Cell experiments have shown that LMHFV regulates the proliferation and differentiation of mesenchymal stem cells by improving gene expressions, growth factor secretion, bone matrix synthesis, energy metabolism etc. (Bacabac et al., 2006; Zhou et al., 2011; Suenaga et al., 2012). This effect was still manifested in implant application by initial stability, biomechanical properties and osseointegration rate of implant, because LMHFV improved promoting bone healing, soft tissue repair and angiogenesis (Shih et al., 2004; Birmingham et al., 2015). However, the transmission mechanism of regulating factors and signaling pathways on LMHFV has not been determined. Further researches on gene expressions are required.

Besides, contact finite element analysis showed different bone responses based on direct forces (i.e., DLV) and indirect forces (i.e., WBV) (Rucci et al., 2007). The main differences between WBV and DLV were shown in Table 3. In particular, WBV had a positive effect during osseointegration process, while DLV only showed a positive effect at the early stage of osseointegration (inflammatory phase and callus formation, for 2 weeks) (Wang et al., 2018; Ruppert et al., 2019).

**Table 3:** Differences between WBV treatment and DLV treatment.

	**WBV treatment**	**DLV treatment**
Mode	General; indirect	Local; direct
Region	Close to the surface of implant	Close to the loading area
Parameters	• *M* = 0.3–0.5 g • *F* = 20–90 Hz • *T* = 15–30 min	• *A* = 5–15 μm • *F* = 20–40 Hz • *T* = 10–20 min
Extent	• Extensive • Whole process of osseointegration	• Circumscribed • Early stage of osseointegration
Strengths	• Definite curative effects • Operating parameters and wide span defined • Result in stochastic resonance and extra bone stimulus	• More suitable for dental implants • Less loading energy • Shorter loading time • Less adverse reactions
Limitations	• Poorly control of the mechanical conditions • Risks of possible adverse reactions from systemic exposure	• Lack of clinical vibration stimulation device • No conclusion on the best parameters
Potential clinical applications	• Bone mineral density in postmenopausal women increases • Adjuvant treatment of osteoporosis • Prevention of falls and fractures in the elderly	• Accelerate implant osseointegration • Promotion of fracture, extraction socket and bone defect healing • Increase bone mass

*A, amplitude (μm); DLV, direct-loading vibration; F, frequency (Hz); M, magnitude (g); T, time (min); WBV, whole-body vibration*.

Moreover, the effect of LMHFV is not as good as that of anti-osteoporosis drugs in OVX animals (Chen et al., 2012). To our interest, the dosage of parathytoid hormonr (PTH) could be reduced appropriately when LMHFV applied simultaneously. This combination could produce synergistic or cumulative effects to some extent (Shibamoto et al., 2018). However, we failed to obtain a pre-conceived better effect of combining LMHFV and alendrinate (ALN), which may be attributed to the inhibited osteoclast activity (Chatterjee et al., 2014). It is suggested that biomechanical interventions can not only improve treatment efficiency, but also reduce drug adverse effects. Thus, researchers can achieve an augmented effect (Shibamoto et al., 2018).

### Clinical Promotion

Compared to WBV loading, DLV loading by small device embedded in mice is more appropriate in oral therapy (Wang et al., 2018), which pronouncedly highlights its convenient clinical application. Differently, WBV loading can play a positive role on systemic diseases treatment (Marín-Cascales et al., 2018), while back pain and Raynaud's syndrome may occur when the body is exposed to an extensive loading for a long time (Yung et al., 2018).

At this stage, LMHFV clinical promotion is limited as follows: (Leung et al., 2014; Wong et al., 2020): (1) Lack of large-scale multi-center clinical RCTs; (2) Unknown exact instruction for jaw bone application; (3) Long treatment period; (4) High medical cost; (5) Lack of follow-up from professional guidance.

### Limitations

Several limitations in our paper should be well-concerned: (1) Among included studies, only one was conducted in jaw bone, and our findings should be fully clinically validated; (2) Due to limited sample size and low evidence level of biomechanical index on osseointegration, it was excluded in our study; (3) Insufficient sample size on osseointegration estimation in other bone diseases, such as bone defect and diabetes; (4) The heterogeneity was unavoidable due to differences between implants and animal species even if we had conducted subgroup analysis; (5) The data conducted in one research team and converted via graphs might lead to potential bias; (6) The quality of evidence was medium or low in this study, the exact conclusion needed to be verified by high-quality studies in the future.

## Conclusion

Based on this systematic review and meta-analysis, LMHFV is demonstrated to be relatively beneficial in improving animal implant osseointegration. The influence factors might be loading parameters and mode. Nevertheless, the fundamental mechanism and ideal parameters remain unclear, which need to be further expounded. Therefore, it is inferred that LMHFV might be a worthy method to improve implant osseointegration clinically, particularly in osteoporosis. Multi-center RCTs with a large sample size are required for examining the efficacy of LMHFV on dental implant clinically.

## Data Availability Statement

The original contributions presented in the study are included in the article/Supplementary Material, further inquiries can be directed to the corresponding author.

## Author Contributions

XY, YB, and SW conceptualized the project. YG, SX, and YZhu conducted the literature review, statistical analyses, and data syntheses. YZha, YL, and YD conducted meta-analyses. JK-H supervised the study. All authors participated in the manuscript preparation with significant intellectual contributions.

## Conflict of Interest

The authors declare that the research was conducted in the absence of any commercial or financial relationships that could be construed as a potential conflict of interest.
