# Supplementary material for: Does Low-Magnitude High-Frequency Vibration (LMHFV) Worth for Clinical Trial on Dental Implant? A Systematic Review and Meta-Analysis on Animal Studies
Source: Front Bioeng Biotechnol. 2021 Apr 27;9:626892. doi: 10.3389/fbioe.2021.626892 (PMC8111077; doi:10.3389/fbioe.2021.626892)

**APPENDIX 1 |** Screening criteria based on the PICOS question.

**Population: Animals (OVX, non-OVX)**

Inclusion:

*(1) Ovariectomized or non-ovariectomized implant osseointegration models of animals;*

*(2) No restrictions on animal species, age and sex;*

*(3) No restrictions on type, specification and implanting-position of implants.*

Exclusion:

*(1) Sample size less than 3 animals;*

*(2) Other implant osseointegration models of animals (e.g. diabetes mellitus models, bone defect models).*

**Interventions: LMHFV (WBV, DLV) loading**

Inclusion:

*(1) Specific regimes satisfies the definition of LMHFV (i.e. LMHFV refers to a vibration wave triggered by low-magnitude (< 1 ×g or <150 μm ) and high-frequency (20-90 Hz));*

*(2) Clear regime vaules (amplitude-μm, magnitude-×g, frequency-Hz) are reported;*

*(3) Specific application means of LMHFV (i.e. Whole-body vibration (WBV) refers to biomechanical loading acted on whole body through trunk to applly an indirect force on the implant; Direct-loading vibration (DLV) refers to biomechanical loading acted on specific parts as needed to creat a direct biomechanical coupling on the implant.)*

Exclusion:

*(1) Parameters used can't conform to the definition of LMHFV;*

*(2) Treatment period is less than 2 weeks.*

**Comparisons: Sham-loading or non-loading**

Inclusion:

*(1) Non-ovariectomized implant osseointegration models: sham-loading/blank control;*

*(2) Ovariectomized implant osseointegration models: sham-loading OVX groups.*

Exclusion:

*Only positive control groups are set.*

**Study design: RCTs studies**

Inclusion:

*Randomized controlled trials (RCTs) of animals.*

Exclusion:

*Not animal RCTs (e.g. clinical trials, cell investigations, reviews, cross-sectional studies, cohort studies, and conference reports).*

**Outcomes: Parameters (magnitude, frequency, duration), efficacy (BIC, BV/TV), gene expression, adverse reactions and related study outcomes;**

Inclusion:

*(1) Investigate the effect of LMHFV on peri-implant bone healing or osseointegration process;*

*(2) Mean BIC (%) and/or mean BV/TV (%) values/charts reported;*

*(3) Types and Regimes of the LMHFV defined;*

*(4) Outcomes related to peri-implant bone morphology/function/adverse reaction/gene express.*

Exclusion:

*Outcomes of the investigations remain unclear or unavailable.*

**Other criteria**

*(1) Full-text literature published in English will be included;*

*(2) Other publication types (reviews, conference reports) will be excluded;*

*(3) Publication period before 15 July, 2020.*

**APPENDIX 2 |** Search protocol used in the systematic review

The following search terms were used for database searches.

**Primary concept**

**Keywords:** *osseointegration*

**Subject headings:**

PubMed/Medline: *"Osseointegration"[MeSH Terms]*

EMBASE: *'osseointegration'/exp OR osseointegration*

OVID: *Osseointegration.mp. [mp=title, abstract, full text, caption text]*

COCHRANE LIBRARY: *Osseointegration OR osseo-integration OR osteointegration OR osseo-integrations*

**Secondary concept**

**Keywords:** *vibration OR mechanical OR (mechanical stimuli) OR (mechanical loading) OR (biomechanical stimuli) OR (biomechanical loading)*

**Subject headings:**

PubMed/Medline: *"Vibration" [MeSH Terms]) OR "Stress, Mechanical" [MeSH Terms]*

EMBASE: *'low magnitude' OR 'high frequency'/exp OR 'high frequency', also uses keywords*

OVID：*(low-magnitude or high-frequency).mp. [mp=title, abstract, full text, caption text], also uses keywords*

COCHRANE LIBRARY: *(low-magnitude) OR (high-frequency), also uses keywords*

**Detailed search queries were as follows:**

MEDLINE/PubMed: *(("Vibration"[Mesh Terms]) OR "Stress, Mechanical"[Mesh Terms]) AND "Osseointegration"[Mesh Terms]*

EMBASE: *('vibration'/exp OR vibration OR 'low magnitude' OR 'high frequency'/exp OR 'high frequency' OR (biomechanical AND stimuli) OR (biomechanical AND ('loading'/exp OR loading)) OR (mechanical AND stimuli) OR (mechanical AND ('loading'/exp OR loading))) AND ('osseointegration'/exp OR osseointegration)*

OVID: *((vibration or low-magnitude or high-frequency or biomechanical stimuli or biomechanical loading or mechanical stimuli or mechanical loading) and Osseointegration).mp. [mp=title, abstract, full text, caption text]*

COCHRANE LIBRARY: *(vibration) OR (low-magnitude) OR (high-frequency) OR (biomechanical stimuli) OR (biomechanical loading) OR (mechanical stimuli) OR (mechanical loading) AND Osseointegration*

**APPENDIX 3 |** Journals included in handsearch.

The journals included in handsearch were as follows:

*Clinical Implant Dentistry and Related Research, Clinical Oral Implants Research, European Journal of Oral Implantology, Implant Dentistry, Implantologie, International Journal of Oral & Maxillo facial Implants, Journal of Oral Implantology, Journal of Periodontal and Implant Science, Journal of Prosthodontics-implant Esthetic and Reconstructive Dentistry, International Journal of Periodontics & Restorative Dentistry, BMC oral Health, Journal of Dental Research, American Journal of Dentistry, Journal of Dental Sciences, Journal of Stomatology Oral and Maxillofacial Surgery.*

**APPENDIX 4 |** Extracted information on study characteristics

Extracted information on study characteristics included publication details (author(s), year of publication), trial study design /study design (controlled versus cross-over, number of experimental groups and subgroups in the experimental groups), source of funding, conflict of interest, study period, and location. Treatment group characteristics included as follows: the study group intervention, including apllication means of LMHFV (WBV and DLV) and regimes of LMHFV (magnitude, amplitude, frequency, duration, interim recovery period or not and other positive intervention or not); comparative group (sham-loading or other control with specification of regimen); type of implant osseointegration animal models (ovariectomized, non-ovariectomized and sham-ovariectomized); the type (rabbit, rat, dog) and other specific information (sex, age and number) of animals; the type (titanium, hydroxyapatite-coated titanium, Ti6Al4V), implanting-position (tibia, jaw, femur) and other specific information (diameter, length and number) of implants. Study outcomes included a summary of outcomes assessed, follow-up times, and study conclusions as well as quantitative results (efficacy of treatment, including BIC and BV/TV) and descriptive results (gene expression and adverse reaction) for outcomes assessed. Extracted data were synthesized in a descriptive table according to the significant.

**APPENDIX 5 |** Reasons for exclusion after the full-text evaluation.

| NO. | Article | Reason for exclusion |
| --- | --- | --- |
| 1 | Hatori et al.(2014) | No implants. |
| 2 | Shi et al.(2010) | No implants. |
| 3 | Matsumoto et al.(2015) | No implants. |
| 4 | Jing et al.(2017) | Other implant osseointegration models of animals. |
| 5 | Jing et al.(2015) | Other implant osseointegration models of animals. |
| 6 | Judex et al.(2009) | Not animal RCTs (review). |
| 7 | Romanos et al.(2004) | Not animal RCTs (review). |
| 8 | Isidor et al.(2006) | Not animal RCTs (review). |
| 9 | Zhao et al.(2009) | Not animal RCTs (hypothesis). |
| 10 | Chen et al.(2016) | Not animal RCTs (cell investigations). |
| 11 | Zhang et al.(2010) | Outcomes of the studies remained unclear or unavailable. |
| 12 | Rubin et al.(1994) | Outcomes of the studies remained unclear or unavailable. |
| 13 | Clark et al.(2005) | Parameters used can't conform to the definition of LMHFV. |
| 14 | Smet et al.(2007) | Parameters used can't conform to the definition of LMHFV. |
| 15 | Duyck et al.(2007) | Parameters used can't conform to the definition of LMHFV. |
| 16 | Matsumoto et al.(2000) | Parameters used can't conform to the definition of LMHFV. |
| 17 | Meyer et al.(2004) | Parameters used can't conform to the definition of LMHFV. |
| 18 | Usui et al.(1989) | Parameters used can't conform to the definition of LMHFV. |
| 19 | Blanco et al.(2013) | Parameters used can't conform to the definition of LMHFV. |
| 20 | Duyck et al.(2006) | Parameters used can't conform to the definition of LMHFV. |
| 21 | Esaki et al.(2011) | Parameters used can't conform to the definition of LMHFV. |
| 22 | Faria et al.(2010) | Parameters used can't conform to the definition of LMHFV. |
| 23 | Jariwala et al.(2016) | Parameters used can't conform to the definition of LMHFV. |
| 24 | Kawahara et al.(2003) | Parameters used can't conform to the definition of LMHFV. |
| 25 | Leucht et al.(2007) | Parameters used can't conform to the definition of LMHFV. |
| 26 | Mehl et al.(2012) | Parameters used can't conform to the definition of LMHFV. |
| 27 | Vandamme et al.(2007) | Parameters used can't conform to the definition of LMHFV. |
| 28 | Vandamme et al.(2008) | Parameters used can't conform to the definition of LMHFV. |
| 29 | Willie et al.(2010) | Parameters used can't conform to the definition of LMHFV. |
| 30 | Yamamoto et al.(2013) | Parameters used can't conform to the definition of LMHFV. |

**APPENDIX 6 |** Assessment of quality and summarizing the findings using the GRADE approach


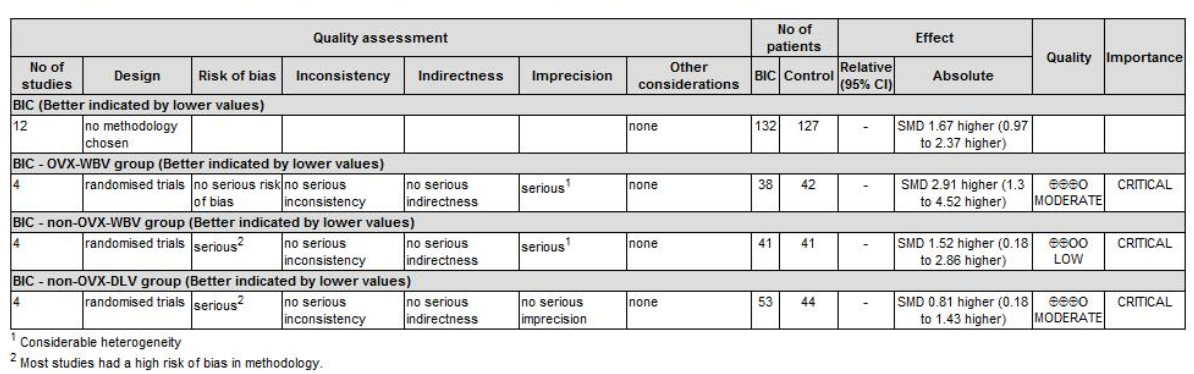

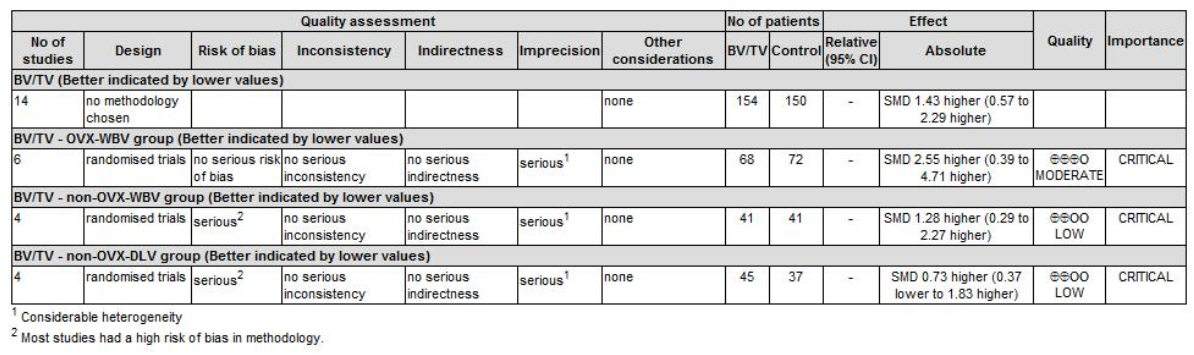

Supplement: Supplementary file 1 [file Data_Sheet_1.docx]
